# Gene expression and nucleotide composition are associated with genic methylation level in *Oryza sativa*

**DOI:** 10.1186/1471-2105-15-23

**Published:** 2014-01-21

**Authors:** Eran Elhaik, Matteo Pellegrini, Tatiana V Tatarinova

**Affiliations:** 1 Department of Mental Health, Johns Hopkins University Bloomberg School of Public Health, 615 N. Wolfe Street, Baltimore, MD 21205 USA; 2 Department of Animal and Plant Sciences, University of Sheffield, Western Bank, Sheffield S10 2TN, UK; 3 Molecular, Cell, and Developmental Biology, University of California, 610 Charles Young Drive East, Los Angeles, CA 90095, USA; 4 Children’s Hospital Los Angeles, Keck School of Medicine, University of Southern California, 4650 Sunset Blvd, Los Angeles, CA 90027, USA

**Keywords:** DNA methylation, Gene expression, GC_3_, Prediction, *Oryza sativa*

## Abstract

**Background:**

The methylation of cytosines at CpG dinucleotides, which plays an important role in gene expression regulation, is one of the most studied epigenetic modifications. Thus far, the detection of DNA methylation has been determined mostly by experimental methods, which are not only prone to bench effects and artifacts but are also time-consuming, expensive, and cannot be easily scaled up to many samples. It is therefore useful to develop computational prediction methods for DNA methylation. Our previous studies highlighted the existence of correlations between the GC content of the third codon position (GC_3_), methylation, and gene expression. We thus designed a model to predict methylation in *Oryza sativa* based on genomic sequence features and gene expression data.

**Results:**

We first derive equations to describe the relationship between gene methylation levels, GC_3_, expression, length, and other gene compositional features. We next assess gene compositional features involving sixmers and their association with methylation levels and other gene level properties. By applying our sixmer-based approach on rice gene expression data we show that it can accurately predict methylation (Pearson’s correlation coefficient r = 0.79) for the majority (79%) of the genes. Matlab code with our model is included.

**Conclusions:**

Gene expression variation can be used as predictors of gene methylation levels.

## Background

Heritable changes in gene expression due to mechanisms other than mutations in DNA sequence are termed “epigenetics”, a term coined in 1957 by Conrad Hal Waddington [1]. These changes are of vast importance to human medical and disease studies. Of all epigenetic mechanisms modulating gene expression, DNA methylation is probably the best understood. Methylation occurs by the addition of a methyl group (−CH3) through a covalent bond to the cytosine bases of the DNA backbone most often at Cytosine-phosphate-Guanine (CpG) dinucleotides [2]. Methylation is common in humans and other mammals, where 70 to 80% of CpG dinucleotides are methylated, yet in some model organisms, such as yeast and fruit fly, there is little or no DNA methylation. Although DNA methylation occurs mostly in the CG context, it may also occur at CHG and CHH sites (where H can be any nucleotide other than G). DNA methylation of CpG dinucleotides is essential for plant and mammalian development, chromosome X inactivation, genomic imprinting, chromosome stability, chromatin structure, the immobilization of transposons, and the control of tissue-specific gene expression [3].

Despite of the importance of methylation to genetic and medical research, the measurement of methylation levels is still not straightforward. Methylation can be detected using a variety of methods such as the sequencing of bisulfite converted DNA, methyl-specific restriction digestion, or immunoprecipitation based approaches [4]. However, these techniques are often laborious and require complex experimental protocols and strict criteria of quality control measures to avoid artifacts and biases. For example, the purity of the chromosomal DNA is crucial for the success of complete bisulfite conversion [5]. PCR is another potential source of artifacts. For example, it could be biased toward amplification of differentially methylated templates if the secondary structure in the amplicon is affected by the presence of guanines or cytosines on either strand [6]. Biases may also occur during amplification of bisulfite-converted DNA and cloning [7]. Clearly, sequence-based techniques would benefit much from reliable computational models. It would therefore be useful to develop computational methods that correctly estimate DNA methylation levels from sequence information.

Due to their great premise, several methods that use sequence data to predict methylation levels were introduced in the past few years. Many of these methods implemented a type of machine learning technique called support vector machine (SVM) (detailed in Fang et al. [8].), which constructs a hyper-plane in a high-dimensional space which is used for classification. One tool that can identify epigenetic modifications is *EpiGRAPH*[9]. *EpiGRAPH* takes into account DNA rise and twist (determining the handedness and pitch of the double helix), frequency of sequence changes (CACC/GGTG, TGTG/CACA, CGCG), and repeat frequencies, and uses an SVM to detect combined effects. Bock et al. [10]. demonstrated that several classes of DNA-related attributes are distinctly associated with CpG island methylation at medium to high rates, such as repeat frequencies and their distributions (with a Pearson’s correlation coefficient of 0.635 and 0.657, respectively). The authors showed that a combination of multiple attribute classes (sequence properties, repeat distribution, gene and exon distribution, SNPs, CpG islands, transcription factor binding sites, and evolutionary conservation) results in a higher correlation value than any single class (0.74).

Based on their observation that methylated and unmethylated sequences differ in the distributions of hexamer motifs that are related to transcription factor binding sites, Das and colleagues [11] developed *HDFINDER*, which achieves 86% accuracy for the prediction of methylation levels. The *MethCGI* tool, developed by Fang and colleagues [8], uses an SVM approach to analyze GC and CpG composition to predict methylation-prone and the methylation-resistant CpG islands. Another tool for the prediction of methylated CpGs in DNA sequences is *Methylator*[12], however Fang and colleagues [8] reported that on HEP data [13] the specificity of and *MethCGI* (38.30%) is higher than that of *Methylator* (21.28%). Most recently, Zhou and colleagues [14] presented another SVM based approach, using a 64-dimensional tri-nucleotide frequency vector to predict CpG methylation in humans, achieving high accuracy of methylation site prediction (the reported accuracy is 0.81).

The broad interest in predicting methylation levels and the heterogeneity of algorithmic approaches illustrate the importance of this problem which often requires adopting different methods for different species. In contrast to many of the previous studies, we focused exclusively on genic regions, as these are often the regions of primary interest. Furthermore, unlike previous studies, we focused on the plant *Oryza sativa*, or rice, which is an important crop and likely representative of monocots. Nonetheless, while our approach is demonstrated on rice, we believe the same methodology could be broadly applicable to related species.

While most previous studies have focused on sequence level determinants to predict methylation, we also decided to look at other factors that may be associated with methylation such as gene expression and its variability across conditions. The motivation to predict DNA methylation from gene expression data and genomic sequences emerges from the observed association between methylation and gene expression [15,16] as well as between methylation levels and CpG content found in previous studies. Genomic regions enriched in CG dinucleotides can, in principle, be preferentially targeted because they provide more substrates for *de novo* methylatrasferases. This targeting, in turn, can affect transcriptional regulation and the variability of gene expression. It seems logical, therefore, that combining our knowledge of sequence compositional features and gene expression could help us better predict DNA methylation levels. Here we introduce a novel approach to predict gene-body methylation in rice using gene expression, GC_3_, and additional compositional features.

## Results

**Figure 1:**
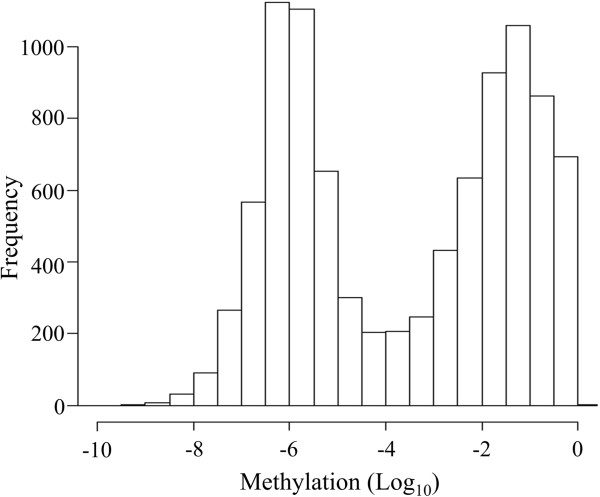
Distribution of gene-body methylation.

**Figure 2:**
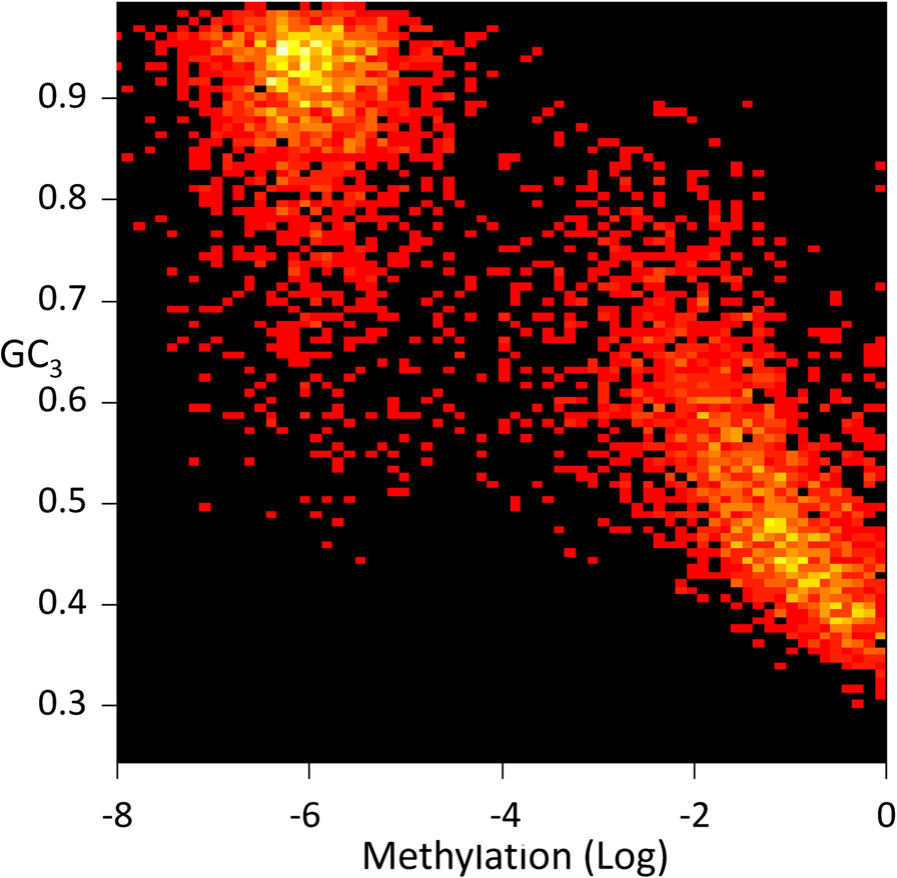
**Log-methylation vs. GC**_**3**_**.**

We first obtained genome-wide DNA methylation data from bisulfite sequence datasets [4], as well as transcriptome data from RNA-seq in rice [17]. Genic methylation levels in rice exhibit a bimodal distribution (Figure 1) dividing genes into highly and lowly methylated groups with a local minimum (“valley”) at 10^-4^. We first studied the relationship between DNA methylation and GC_3_, the GC content of the third codon, a metric that we have extensively studied in the past [18-20]. *O. sativa* has two distinct classes of genes (GC_3_-rich and -poor) (Additional file 1: Figure S1). We found that the methylation of unmethylated genes (with a methylation level < 10^-4^) is not correlated with GC_3_ (*r* = 0.08). By contrast, the methylation level of methylated genes (with a methylation level ≥10^-4^) is negatively correlated with GC_3_ (*r* = −0.68) Figure 2, Table 1).

**Table 1:** Correlation between gene-body methylation and other genic features

**Parameter**	**Unmethylated**	**Methylated**
GC_3_	0.08	−0.68
Mean expression	0.07	0.22
Standard deviation of gene expression	0.14	0.06
Coefficient of variation of gene expression	−0.04	−0.18
Relative abundance of CpG	0.07	−0.70
Gene length	0.06	0.29

**Figure 3:**
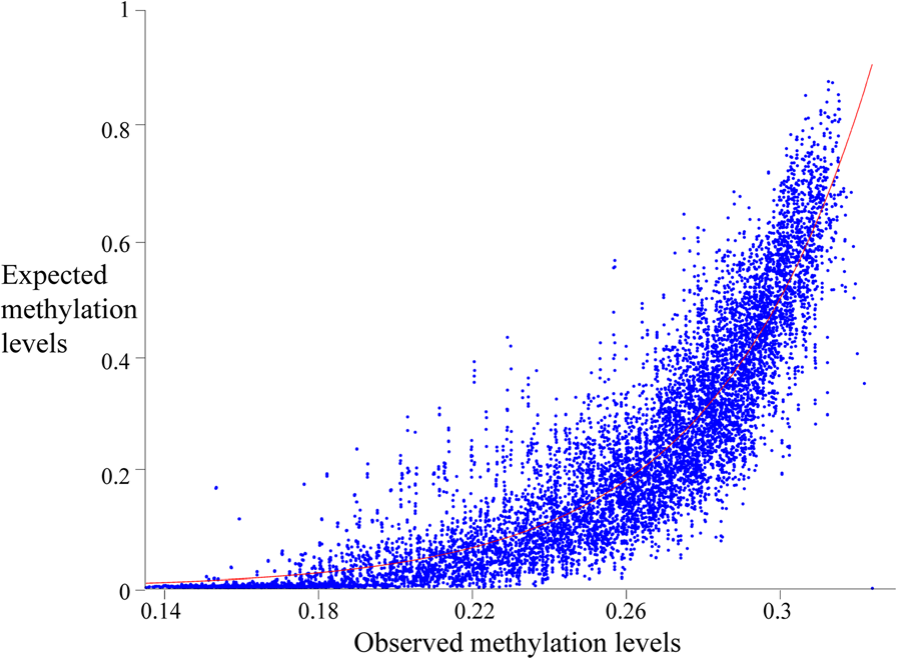
**Gene methylation levels estimated from sixmers (x-axis) and the gene methylation level obtained from experimental data (after moving average smoothing).** An exponential fitting is shown in red.

To identify other factors that are associated with DNA methylation, we calculated Pearson correlation coefficients between nucleotide compositional features as well as gene expression and methylation in *O. sativa* (Table 2). Many of these gene level measurements have modest levels of association with DNA methylation. To identify if higher resolution properties of genes are more strongly associated with methylation, we also computed sixmers along coding regions. The choice of sixmers was a compromise between the need to capture the genomic variation between adjacent codons and minimizing the number of possible *n*-mers. On average, each of the 4,096 (4^6^) possible sixmers appears 4,240 times across rice genes. The methylation level per sixmer type was calculated as a weighted average, based on the frequency of that sixmer in all genes (*m*):

(1) $$\mathrm{Met}\;\left(\mathrm{sixmer}\right)=\frac{\sum_{i=1}^{m}\mathrm{Met}{\left(\mathrm{gene}\right)}_{i}\times n{\left(\mathrm{sixmer}\right)}_{i}}{\sum_{i=1}^{m}n{\left(\mathrm{sixmer}\right)}_{i}},$$

where *Met*(*gene*)_*i*_ is the methylation level in gene *i* and *n*(*sixmer*)_*i*_ is the number of occurrences of a *sixmer* type in gene *i*. This analysis yielded a vector of 4,096 possible sixmers and their associated methylation levels (see Additional file 2). To confirm that sixmer methylation levels are a good model for gene methylation we calculated the methylation level per gene, so that

(2) $$\mathrm{Met}{\left(\mathrm{gene}\right)}^{'}=\frac{\sum_{\mathrm{sixmer}=1}^{4,096}\mathrm{Met}\left(\mathrm{sixmer}\right)\times n\left(\mathrm{sixmer}\right)}{l},\mathrm{Met}\left(\mathrm{sixmer}\right)>0$$

where *Met(sixmer)* is the methylation level per sixmer type, *n(sixmer)* is the number of the sixmer copies in the gene, and l is the gene length. The relationships between these methylation values to the expected methylation values, after using moving average fitting, follows an exponential distribution (*r*^2^ = 0.83, SSE = 82) (Figure 3):

(3) $$\mathrm{Met}\left(\mathrm{gene}\right)=6\cdot 10^{-4}e^{21.33\cdot \mathrm{Met}{\left(\mathrm{gene}\right)}^{'}}.$$

**Table 2:** **Correlation ( *r *) between nine gene compositional features and gene body-methylation**

**Variable**	**Short name**	**R**
GC_3_	GC_3_	−0.673
Gene expression: mean (*μ)*	GE_MEAN	0.255
Standard deviation (*σ)*	GE_STDEV	0.084
CV of expression ($\frac{\sigma }{\mu }$)	GE_CV	−0.217
Genome signature (*ρ*= $\frac{f_{\mathrm{CG}}}{f_{C}f_{G}}$)	GEN_SIG	−0.697
CDS length	l	0.286
Change in CG_3_ from the left to the middle of the gene	GRADLM	0.269
Change in CG_3_ from the middle to the of the right gene	GRADMR	−0.289
CG_3_ in the left third of the coding sequence (CDS)	GCL	−0.364
CG_3_ in the middle third of CDS	GCM	−0.545
CG_3_ in the right third of CDS	GCR	−0.343

We thus confirmed that methylation measured using sixmers is monotonically associated with the observed gene methylation.

Due to this strong association between the methylation of sixmers and that of genes, we sought to predict sixmer methylation using gene level properties other than methylation itself. To model the relationships between sixmer methylation and different genomic features, we first computed for each gene the GC_3_; genome signatures $\rho_{\mathrm{CG}}=\frac{f_{\mathrm{CG}}}{f_{C}f_{G}}$ (GEN_SIG), GC_3_ levels along the first (GCL), second (GCM), and the last thirds of the gene (GCR), gene length (*l*), and GC_3_ gradient from left to the middle (GRADLM) and from the middle to the right (GRADMR). Gene expression average (GE_MEAN) and standard deviation (GE_STD) were obtained from public databases (see *Methods*) and were used to calculate the coefficient of variation of gene expression (GE_CV) (Table 2).

We used Eq. 1 to calculate the weighted average of each genomic feature per sixmer, by replacing *Met* with that feature. For example, for GE_CV(sixmer) we have the following equation:

(4) $$\mathrm{GE}\_\mathrm{CV}\left(\mathrm{sixmer}\right)=\frac{\sum_{i=1}^{m}\mathrm{GE}-\mathrm{CV}{\left(\mathrm{gene}\right)}_{i}\times n{\left(\mathrm{sixmer}\right)}_{i}}{\sum_{i=1}^{m}n{\left(\mathrm{sixmer}\right)}_{i}},$$

To reduce the number of variables in our analysis, we first calculated the standard deviation of each genomic feature per sixmer type. We observed a wide dispersal of the standard deviations. We adopted a 0.1 cutoff for the standard deviation and excluded gene compositional features whose median of the standard deviation was above this cutoff (Additional file 1: Figure S2) leaving only two gene expression features: GE_STD, GE_CV (Table 2). We found an extremely high correlation between methylation and each of these two summaries of gene expression (*r* = 0.95) across all sixmer types. We then tested the ability of each feature to predict the methylation level by splitting the original dataset into half and using cross validation to test the two features together and separately. We found that GE_CV alone fit the model best and derived a linear regression with the following coefficients:

(5) $$\begin{array}{l} \mathrm{Met}\left(\mathrm{sixmer}\right)=-1.66\times \mathrm{GE}\_\mathrm{CV}\left(\mathrm{sixmer}\right)\\ \;+1.7567 \end{array}$$

which was found to explain the methylation levels in the second dataset very well (*r* = 0.82, *p* < 10^-16^) (Additional file 1: Figure S2).

These results show that for each sixmer type, GE_CV, the coefficient of variation of gene expression, is strongly correlated with methylation. Therefore, by knowing the gene’s coefficient of variation of expression level, we can calculate the coefficient of variation for each sixmer type, using Eq. 4. Next, we can use Eq. 5 to calculate the predicted methylation level for each sixmer, and finally use Eqs. 2 and 3 to predict the methylation level of the gene. We emphasize the importance of using sixmers in our approach as the gene methylation and coefficients of variation for gene expression are not correlated (*r* = 0.083).

**Figure 4:**
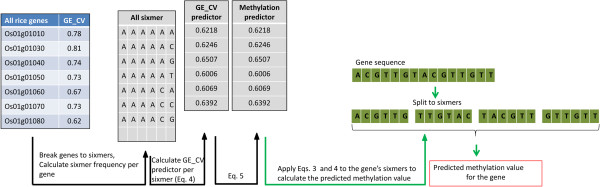
**A flow chart of the proposed algorithm to calculate methylation from gene expression data (left to right).** Calculations marked in black arrows are carried once for all genes, whereas calculation marked in green are carried for each specific gene.

The complete algorithm is illustrated in Figure 4 and by the following example. Consider the gene *Os01g01040,* which has 1,575 nucleotides that can be classified into 1,783 sixmer types. To predict its methylation level, we first used Eq. 4 to calculate GE_CV for all sixmer types using GE_CV of all rice genes. We then used Eq. 5 to predict the methylation level for each sixmer. These steps are not gene-specific and need to be carried out only once. Considering the particular sixmers of our gene of interest, we used Eqs. 2 and 3 to calculate the weighted average of the predicted methylation level for this gene. The predicted gene methylation level of 0.21 was only 0.02 lower than the actual methylation level.

Overall, the correlation between our predicted methylation values and gene methylation was *r* = 0.74 (Additional file 1: Figure S3). This correlation is slightly lowered by a large number of outliers and genes with low GE_CV that are poorly predicted (Additional file 1: Figure S4). We speculate that these outliers may be due to sequencing errors. We identified a large gene subset (79% of the genes) that exhibit higher correlation (*r* = 0.79, n = 9,547). Genes with low GRADLM (≤0.07) and GRADMR (≤0.2) had also higher correlation (*r* = 0.79, n = 4,386) as well as long genes (*l* > 900nt, *r* = 0.76, n = 8,646) (see Table 2). We thus demonstrated that gene expression in rice is a useful biomarker to predict gene methylation levels most accurately for the vast majority of the genes.

We note that the two well-studied gene compositional features genome signature (CpG Expected/Observed) and GC_3_ were excluded from our model due to their high mean and standard deviation per sixmer type (Additional file 1: Figures S2). To illustrate the effect of this deviation on the results we attempted to develop a GC_3_-based model. Although sixmer GC_3_ alone appears to be highly correlated with the sixmer methylation levels (*r* = 0.97, *p* < 10^-16^), the correlation with the test dataset was lower (*r* = 0.88) and the confidence intervals were two orders of magnitude larger than in the above derivation. Overall, our results show that sixmer and sequence compositional features are strongly correlated with sixmer methylation levels and can be used as a marker to predict gene methylation.

The influence of the number of experiments onto prediction accuracy was analyzed using data from 107 experiments (obtained from NCBI’s GEO datasets GSE26280, GSE6737, GSE5167, GSE4438, and GSE7951). The number of experiments necessary to reduce the error in the GE_CV is illustrated in Additional file 1: Figure S5. In this figure, we see that *N = 20* experiments are sufficient to determine CV with approximately 20% precision. Precision for *N* experiments is calculated as *Error*(*N*) = [*abs*(*CV*(*N*) − *CV*(107))/*CV*(107)] × 100*%*, where we assume that the asymptotic value of CV is achieved at 107 experiments. The low costs of microarrays and the wealth of existing gene expression data in public datasets suggest that our proposed solution is both financially plausible and applicable.

## Discussion

Knowledge of methylation levels of genes is important for understanding gene regulation and gene expression. DNA methylation is currently being detected mostly by experimental methods that are laborious and expensive and may be inaccurate, which necessitate the development of computational prediction methods. Although such methods would not be able to predict changes in methylation due to developmental or environmental effects, they can be used to test the accuracy to sequence-based approaches and might be able to infer the predisposition of various genes to be methylated.

In our earlier study [20], we have analyzed the relationship between DNA methylation and alternative splicing in rice and three other taxa. We have shown that compositional features are correlated with methylation levels and proposed that the relationship between GC content measures, methylation, and expression patterns may be utilized to infer one from the others. Here, we propose a novel approach to infer methylation levels from sequence and expression data and we demonstrate its applicability to *O. sativa* genes.

We found that while many gene level properties are correlated with methylation, we could considerably improve our predictive power by considering a gene as an ensemble of sixmers. Our approach is to study gene expression data to estimate the methylation of sixmers which are then used to predict methylation levels of genes. We find that this approach is highly accurate (*r* = 0.79) for the vast majority of the genes (79%). In particular, we found that the coefficient of variation of gene expression by itself allows us to accurately model the methylation of a gene. However, while the direct association between gene level methylation and the coefficient of variation of gene expression is weak, when we first compute this parameter for sixmers and then infer the level of methylation for genes based on their sixmer content we achieve dramatically better results.

It is not surprising that the methylation of a gene is strongly associated with the variation of its expression across multiple datasets. We have previously shown that the two properties are associated in rice as well as other organisms [19,20]. Nonetheless, it is remarkable that the methylation of a gene can be so accurately captured based on its sixmer content and their associated expression variation. This conclusion underlines the strong association between methylation and gene expression regulation. This conclusion supports extensive prior studies suggesting that methylation is an important tissue specific regulatory mechanism.

This study focused on the variation of gene expression across all tissue types, developmental stages, and external conditions; however, our framework can be applied to the analysis to the environmental perturbation-associated variability by calculating the variance of gene expression using external stimuli. With the increased availability of RNA sequencing based gene expression data, our approach can be seamlessly extended to predict exon-level DNA methylation signatures which may be useful for detection and interpretation of alternative splicing events.

## Conclusions

In this paper, sequence compositional features and gene expression were utilized to develop a model for the prediction of gene-body methylation in rice. Our results indicate that the proposed method has the ability to achieve accurate prediction of methylation based exclusively on gene expression. These results suggest that gene body methylation is strongly associated with the variation of the expression of genes across multiple conditions.

## Methods

### Data

Gene models for *Oryza sativa* ssp *japonica* were taken from MSU (version 6.1); Gene-body methylation bisulfite sequencing measurements were obtained from previous studies [4]. In order to call methylation at a single site we required to have a minimum of five reads. In bisuflite sequencing, DNA methylation is defined as the fraction of cytosines that failed to undergo bisulfite convergence. Therefore, for each cytosine, the methylation level ranges from 0 to 1. When we compute average gene-body methylation across all exonic regions. The proposed framework can be extended to other species.

### Gene expression data

Mean, standard deviation and coefficient of variation for gene expression were computed across microarray experiments, obtained from NCBI GEO (GSM404358, GSM404359, GSM404360, GSM404361, GSM404362, GSM404363, GSM404364*,* GSM404365*,* GSM404366*,* GSM404367*,* GSM404368*,* GSM404369*,* GSM404370*,* GSM404371*,* GSM404372*,* GSM404373*,* GSM404374*,* GSM404375*,* GSM404376*,* GSM404377*,* GSM404378*,* GSM404379, GSM183474*,* GSM183475*,* GSM183476*,* GSM183477*,*GSM183478*,* GSM183479*,* GSM183480*,* GSM183481*,* GSM404380, and GSM404381).

Mean gene expression for each gene *g* is calculated as: $\mathrm{GE}\_\mathrm{MEA}N_{g}=\frac{\sum_{i=1}^{N}\mathrm{GE}\left(g,i\right)}{N}$, where N is a number of microarray experiments and *GE*(*g*, *i*) is expression of gene *g* in experiment *i*.

Standard deviation of gene expression for each gene *g* is calculated as: $\mathrm{GE}\_\mathrm{ST}D_{g}=\sqrt{\frac{\sum_{i=1}^{N}{\left(\mathrm{GE}\left(g,i\right)-\mathrm{GE}\_\mathrm{MEA}N_{g}\right)}^{2}}{N}}$, where N is a number of microarray experiments and *GE*(*g*, *i*) is expression of gene *g* in experiment *i*.

Coefficient of variation of gene expression for each gene *g* is calculated as: $\mathrm{GE}\_CV_{g}=\frac{\mathrm{GE}\_\mathrm{ST}D_{g}}{\mathrm{GE}\_\mathrm{MEA}N_{g}}$.

### Data analysis

To study the relationship between methylation and gene expression we selected a dataset of 13,471 genes that have full-length cDNA support, do not encode transposable elements, and have gene expression data, as well as reliable gene models and methylation data (see Additional file 3).

We identified eleven sequence compositional features and tested their ability to predict gene-body methylation (Table 2). Few of these features were previously shown to be related to methylation (e.g., [21]) but most are unique to our study.

### Prediction validation

There are four general methods of validation of regression model: (i) Comparison of the model predictions and coefficients with physical theory; (ii) Collection of new data to check model predictions; (iii) Comparison of results with theoretical models and simulated data; and (iv) separation of data into testing and training sets to generate an independent measure of the model prediction accuracy [21]. Lack of theory leaves us with only one option (iv). The entire dataset was divided into two subsets of approximately equal size. Sixmers were obtained from the coding regions of genes by concatenating every two adjacent codons. We examined 8.8 million sixmers that comprise the coding regions of the 13,471 selected rice genes (see Additional file 3). The linear model was fit using the multiple linear regression *regress()* function in Matlab.

### Files and code availability

Matlab code and Additional files 2 and 3 are available from https://code.google.com/p/methylationpredictor/.

## Competing interests

The authors declare that they have no competing interests.

## Authors’ contributions

TT and EE designed the study and carried out all analyses. MP conceived of the study, and participated in its implementation. All authors were involved in preparation of the manuscript; they read and approved the final version of it.

## Authors’ information

EE was a research associate in the Department of Mental Health, Johns Hopkins University Bloomberg School of Public Health. Currently, EE is a lecturer at the Department of Animal and Plant Sciences, The University of Sheffield, UK.

MP is a professor at Department of Molecular, Cell and Developmental Biology, UCLA.

TT is an associate professor at the Children’s Hospital Los Angeles, Keck School of Medicine, University of Southern California, USA.

## Supplementary Material

Additional file 1: Figure S1 *O. sativa* distribution of GC_3_ in coding sequences. **Figure S2**: The mean (top) and standard deviation (bottom) of a dozen gene compositional features calculated across all 4,096 sixmers. **Figure S3**: Coefficient of variation of gene expression for well and poorly predicted genes. **Figure S4**: Linear regression between observed (x-axis) and expected (y-axis) methylation levels per 13,471 genes. The linear fitting line is marked in red. Each dot represents a gene. **Figure S5**: Estimation of the error rate in the coefficient of variation of gene expression per number of experiments. Click here for file

Additional file 2 **The mean methylation, *GC***_***3***_, ***GE_MEAN***, ***GE_STDEV, GE_CV, GEN_SIG, GCL, GCM, GCR, l, GRADLM*, and *GRADMR *** **for each sixemr type when averaged over rice genes.** Click here for file

Additional file 3 Genomic sequence features and gene expression statistics for 13,471 rice genes. Click here for file
