# Supplementary material for: Gene expression and nucleotide composition are associated with genic methylation level in Oryza sativa
Source: BMC Bioinformatics. 2014 Jan 21;15:23. doi: 10.1186/1471-2105-15-23 (PMC3903047; doi:10.1186/1471-2105-15-23)

Gene expression and nucleotide composition are associated with genic methylation level in *Oryza sativa*

Eran Elhaik^1^, Matteo Pellegrini^2^ Tatiana Tatarinova ^3,4*^

Department of Mental Health, Johns Hopkins University Bloomberg School of Public Health, Baltimore, MD, USA

Molecular, Cell, and Developmental Biology, University of California, Los Angeles, Los Angeles, CA, USA

Glamorgan Computational Biology Research Group, University of Glamorgan, Wales, United Kingdom

Laboratory of Applied Pharmacokinetics, University of Southern California, Los Angeles, CA, 90089, USA

**Supplementary Figures**

Figure S1: *O. sativa* distribution of GC_3_ in coding sequences


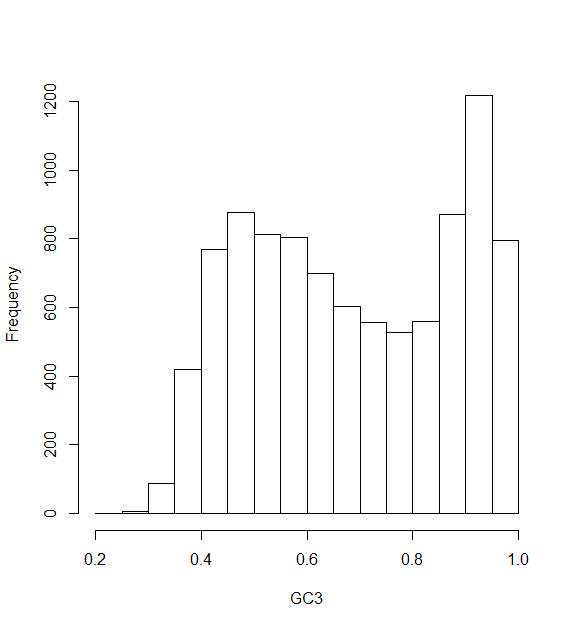


Figure S2: The mean (top) and standard deviation (bottom) of a dozen gene compositional features calculated across all 4,096 sixmers.


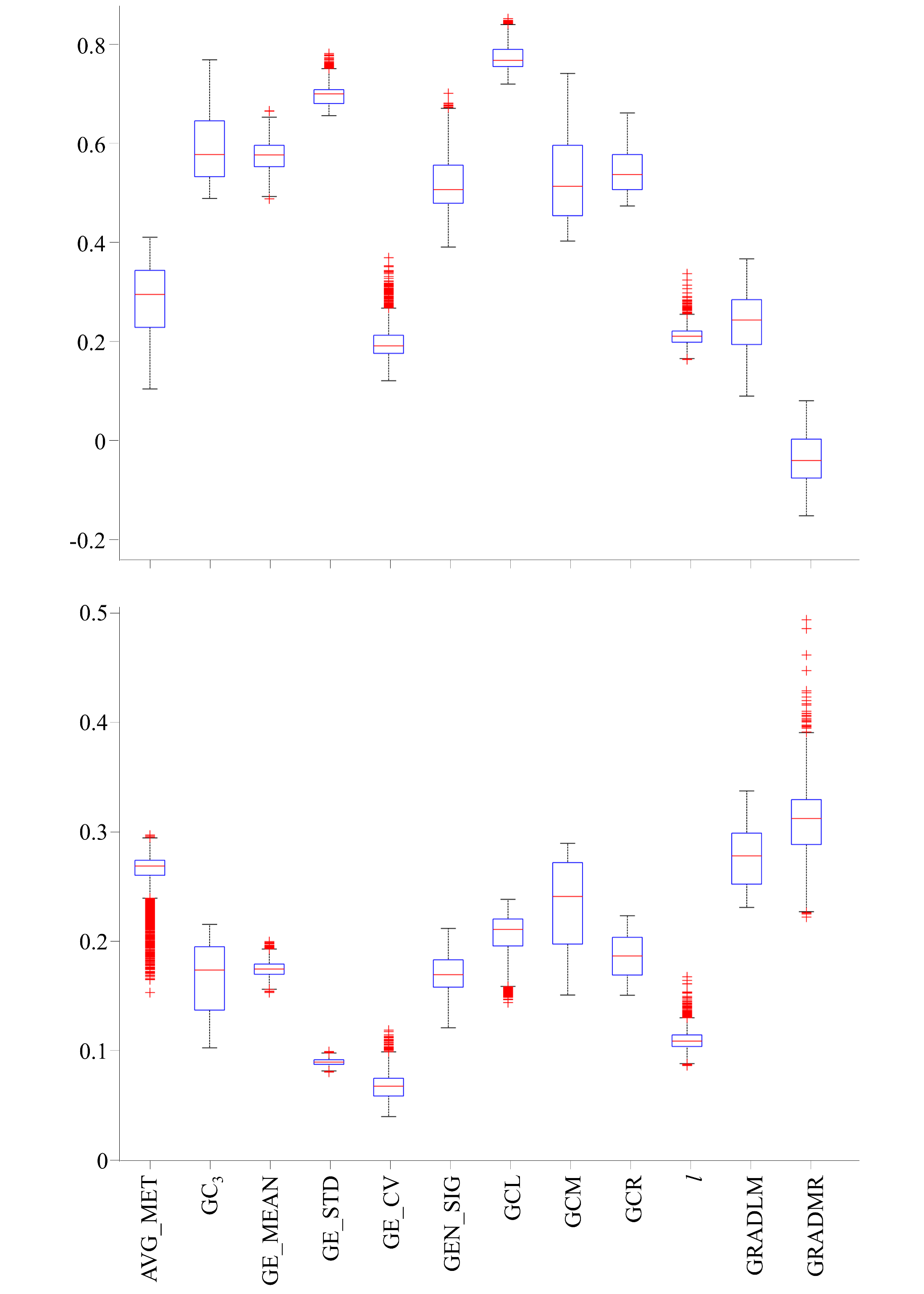


Figure S3: Coefficient of variation of gene expression for well and poorly predicted genes


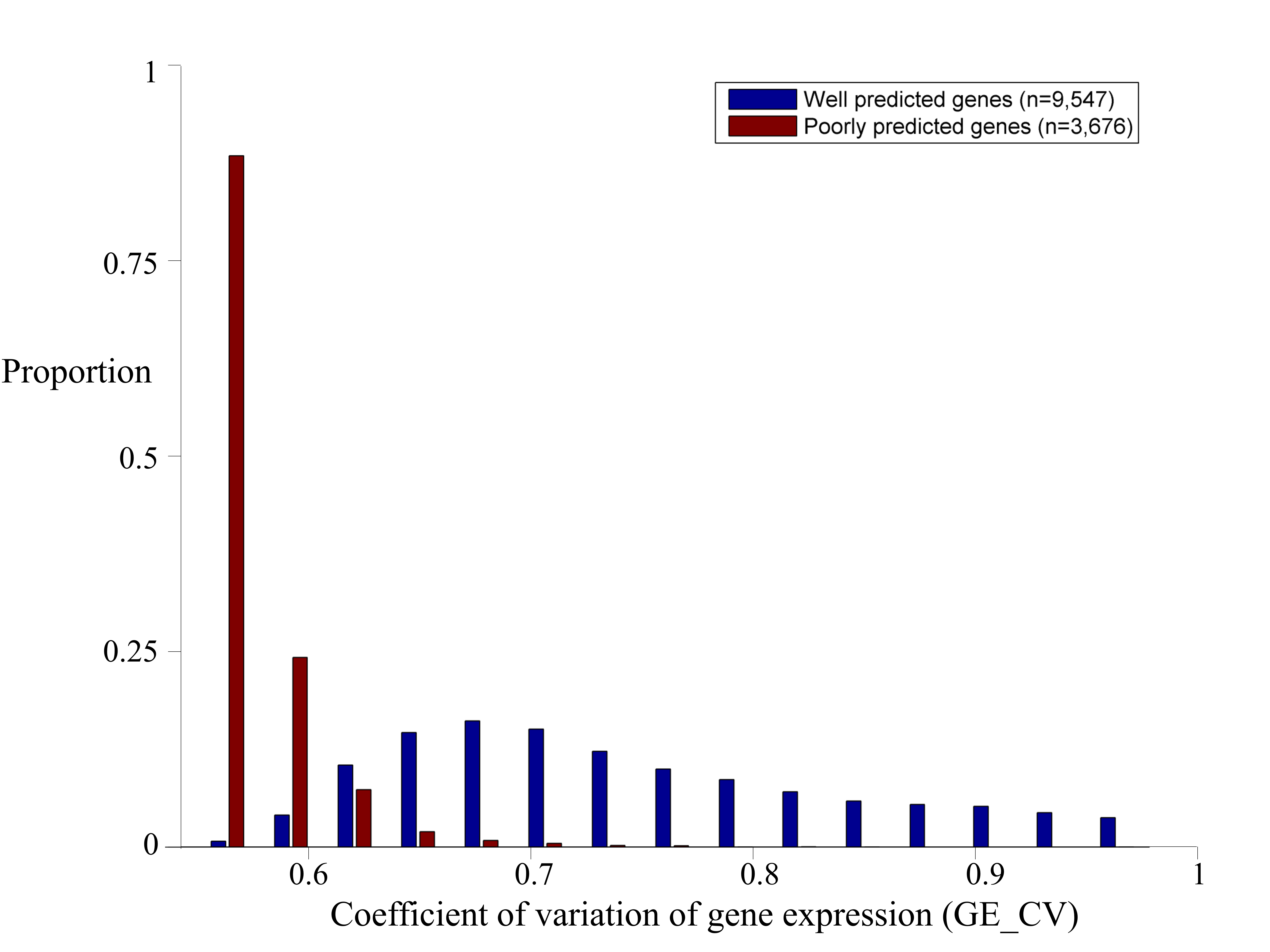


Figure S4

Linear regression between observed (x-axis) and expected (y-axis) methylation levels per 13,471 genes. The linear fitting line is marked in red. Each dot represents a gene.


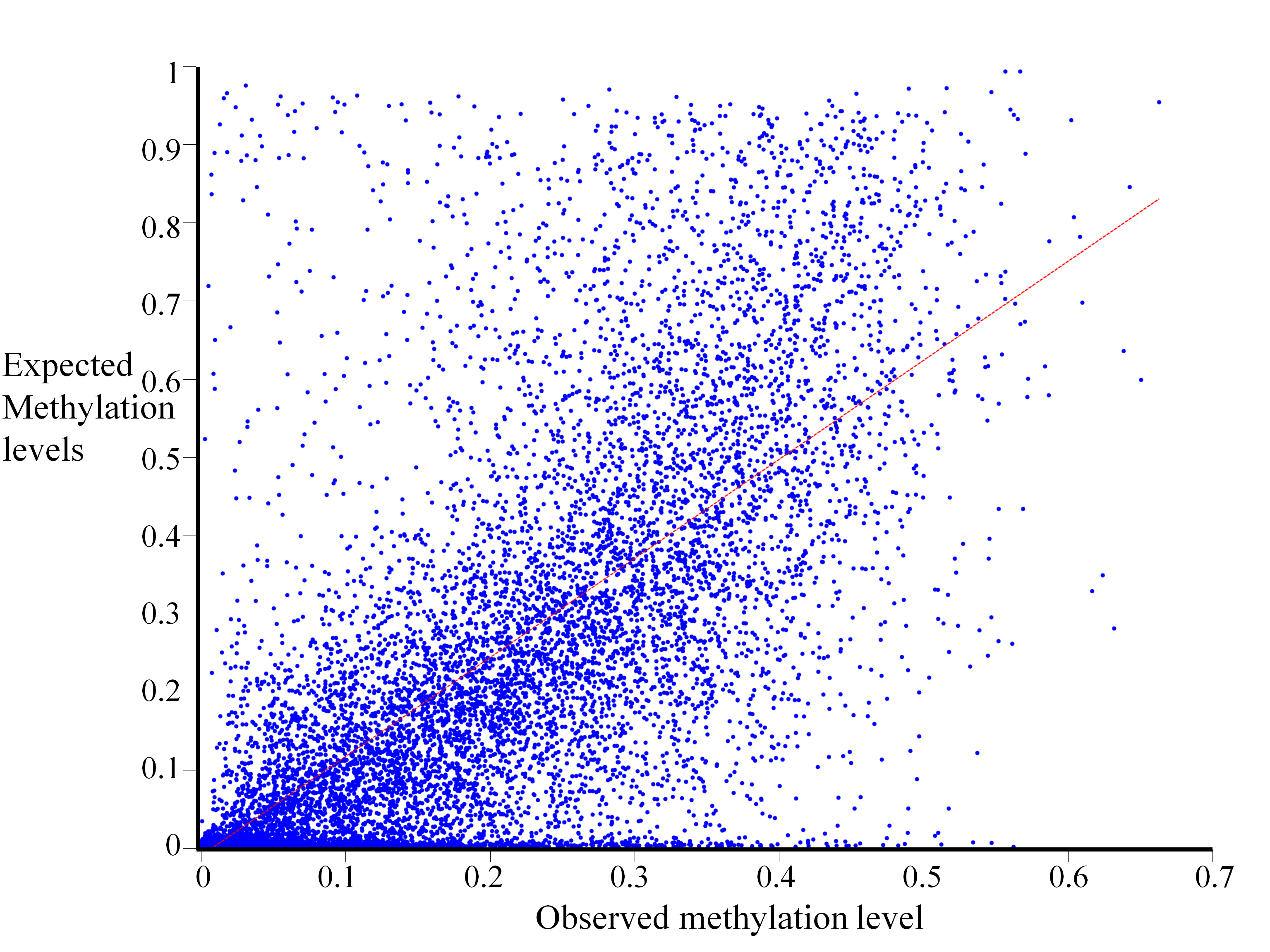


Figure S5

Estimation of the error rate in the coefficient of variation of gene expression per number of experiments.


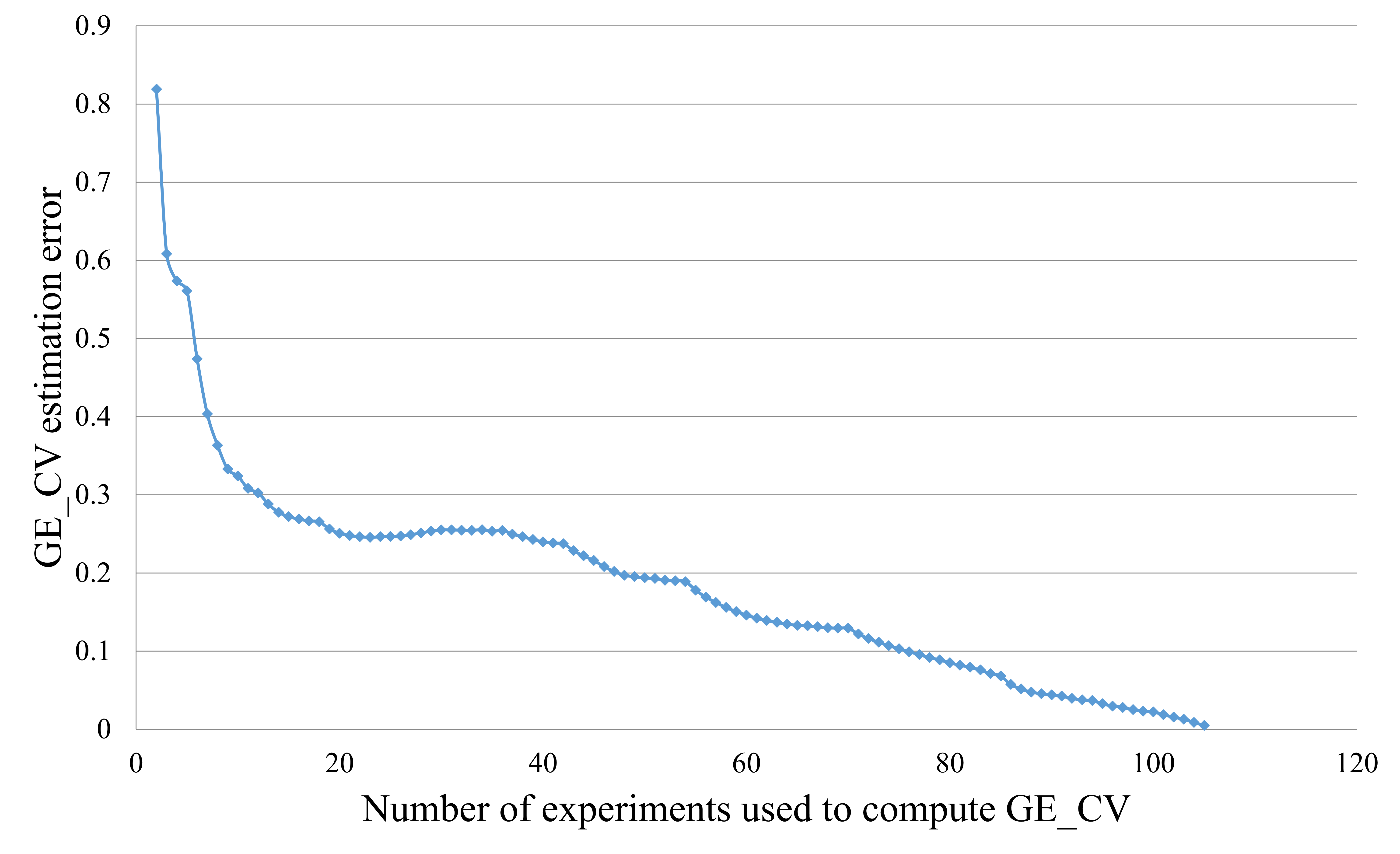

Supplement: Additional file 1: Figure S1 — O. sativa distribution of GC3 in coding sequences. Figure S2: The mean (top) and standard deviation (bottom) of a dozen gene compositional features calculated across all 4,096 sixmers. Figure S3: Coefficient of variation of gene expression for well and poorly predicted genes. Figure S4: Linear regression between observed (x-axis) and expected (y-axis) methylation levels per 13,471 genes. The linear fitting line is marked in red. Each dot represents a gene. Figure S5: Estimation of the error rate in the coefficient of variation of gene expression per number of experiments. [file 1471-2105-15-23-S1.docx]
